# Current methods for contactless optical patient diagnosis: a systematic review

**DOI:** 10.1186/s12938-023-01125-8

**Published:** 2023-06-17

**Authors:** Belmin Alić, Tim Zauber, Christian Wiede, Karsten Seidl

**Affiliations:** 1 Department of Electrical Engineering and Information Technology, University of Duisburg-Essen, Bismarckstr. 81, 47057 Duisburg, Germany; 2 Department of Embedded Software and Embedded AI, Fraunhofer Institute for Microelectronic Circuits and Systems, Finkenstr. 61, 47057 Duisburg, Germany; 3 Business Unit Health, Fraunhofer Institute for Microelectronic Circuits and Systems, Finkenstr. 61, 47057 Duisburg, Germany

**Keywords:** Review, Contactless, Optical, Vital signs, diagnosis

## Abstract

Many countries around the world face a shortage of medical personnel, leading to work overload or even burnout. This calls for political and scientific solutions to relieve the medical personnel. The measurement of vital signs in hospitals is still predominately carried out manually with traditional contact-based methods, taking over a substantial share of the medical personnel’s workload. The introduction of contactless methods for vital sign monitoring (e.g., with a camera) has great potential to relieve the medical personnel. This systematic review’s objective is to analyze the state of the art in the field of contactless optical patient diagnosis. This review distinguishes itself from already existing reviews by considering studies that do not only propose the contactless measurement of vital signs but also include an automatic diagnosis of the patient’s condition. This means that the included studies incorporate the physician’s reasoning and evaluation of vital signs into their algorithms, allowing an automated patient diagnosis. The literature screening of two independent reviewers resulted in a total of five eligible studies. The highest number of studies (three) introduce methods for the risk assessment of infectious diseases, one study introduces a method for the risk assessment of cardiovascular diseases, and one study introduces a method for the diagnosis of obstructive sleep apnea. Overall, high heterogeneity in relevant study parameters is reported among the included studies. The low number of included studies indicates a large research gap and emphasizes the demand for further research on this emerging topic.

**Supplementary Information:**

The online version contains supplementary material available at 10.1186/s12938-023-01125-8.

## Introduction

Many countries around the world face shortage of medical personnel, such as Germany [1] or the UK [2]. The COVID-19 pandemic has emphasized this issue even further and led to appeals from medical personnel associations to their governments to take action [3]. The staff shortage leads to work overload and ultimately burnout [3]. For the scientific community, these issues rise the need for designing solutions that will relieve the medical personnel. The frequent, however necessary, measurement of vital signs takes up a large portion of the medical personnel’s workload. A study has shown that on average it takes 6.5 min per patient for one measurement [4]. Regardless, the majority of vital sign measurements are still carried out manually with traditional contact-based methods [5]. Contact-based measurements encompass a number of disadvantages, starting with the high workload for the staff and continuing with a higher risk of a viral transmission [6–10], patient discomfort, reduced mobility, risk of skin irritation [11] and risk of measurement deviations [12]. The aforementioned disadvantages may be reduced using contactless measurement methods. These methods reduce the contact between the medical staff and the patients, thereby reducing the risk of viral transmission and reducing the staff’s workload. Besides the benefits for the medical staff, contactless methods enhance patient comfort by removing the risk of skin irritation and mobility constraints [11].

Most contactless methods are based on optical and radar technology, but there are also approaches using Wi-Fi, RFID, and acoustics [5]. In the scope of this review, only optical methods are considered. This boundary is set due to the following reasons: (1) optical and radar technologies are currently the two most advanced and most promising contactless patient monitoring technologies [5]; (2) the capabilities of radar-based monitoring are already discussed in [13]; and (3) no previous review on the same topic was found. Several review articles on the topic of contactless optical vital signs monitoring already exist, such as [14, 15]. This systematic review focuses on research publications that not only propose contactless optical measurements of vital signs but also provide a fully automated diagnosis of the patient’s condition. To clarify the focus of this article, the following example is provided. In article number one [16], a research group introduces an algorithm to measure heart rate and respiration rate using an infrared camera. In article number two [17], the same group extends their work using the measured vital signs to autonomously diagnose a person with an infectious disease. Article number one is not included in this review, while article number two is.

The goal of this article is to analyze the state of the art in the topic of fully autonomous patient condition diagnosis, observe the use cases for which these studies have been proposed, compare the used algorithms and classification methods, compare the hardware setups, give a discussion on the current state of technology and state the challenges and the potentials for future work in this field. The structure of the article is the following: (1) a brief introduction to the topic and motivation for the review; (2) results of the review; (3) discussion; (4) conclusion; and (5) a statement of the methods used in the review process.

## Results

### Study selection

**Fig. 1:**
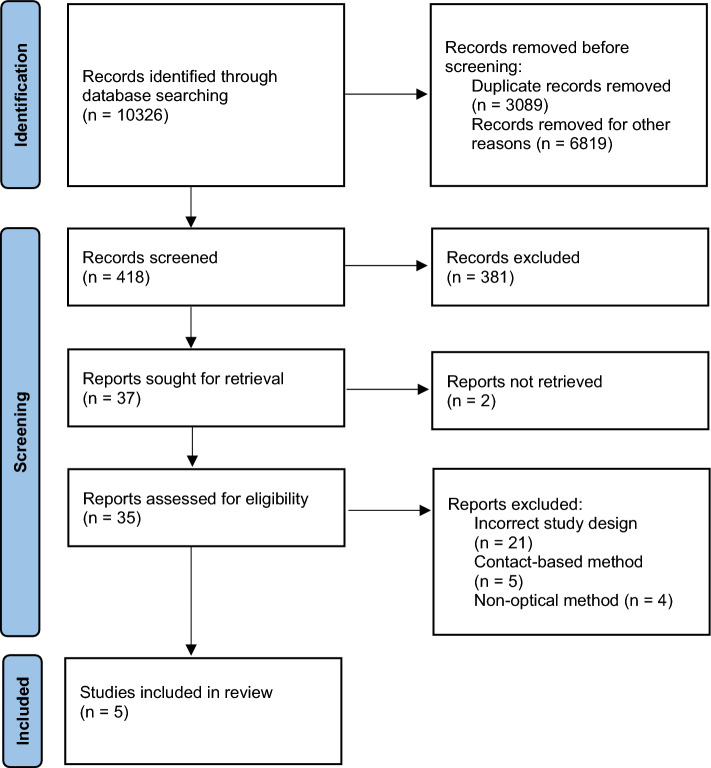
PRISMA flow diagram of the study selection process

The search yielded 10,326 articles. After removing the duplicates and irrelevant search results, the titles and abstracts of a total of 418 articles are screened. Out of the 418 screened articles, 381 are directly excluded for failing to meet the eligibility criteria and 37 are sought for retrieval. Out of the 37 articles, 35 are obtained and two were not retrieved. The 35 obtained articles are assessed in detail according to the eligibility criteria. This resulted in five articles being included in the study and 30 articles being excluded for not meeting the eligibility criteria. Out of the 30 excluded articles, 21 are excluded due to an incorrect study design (e.g., for not incorporating a diagnosis of the patient condition, but rather only measuring one or multiple vital signs), five are excluded for containing contact-based methods and four are discarded for containing non-optical technologies (e.g., radar or wearables). It is to be noted that one study [18] does not provide a final diagnosis, but rather discusses the benefits it brings to support the patient diagnosis. The reviewers decided to include this study in the review even though the eligibility criterion (3) is not fully met, because this study introduces a new use case for patient diagnosis and a clear statement of how their work will benefit the diagnosis process. The PRISMA flow diagram of the study selection process is shown in Fig. 1. A detailed protocol of records identification, screening and decisions is provided in Additional file 1.

### Summary of included studies

In the work of Sun et al. (2017) [17], a combined infrared thermography (IRT) and RGB camera system are developed to screen patients with suspected infectious diseases by measuring the heart rate (HR), respiratory rate (RR), and body temperature (BT) and performing a logistic regression discriminant analysis. The measurement hardware included a 0.3 MP IRT camera and a 0.3 MP CMOS RGB camera. A single rPPG signal from an ROI on the forehead is computed from the green channel of the RGB camera for obtaining the HR. The RR is computed by analyzing relative temperature variations in the nasal area via the IRT camera. The BT is determined by facial skin temperature measurement via the IRT camera. The vital sign computation is presented in detail in a previous publication of the same research group in [16]. The computed three vital signs are used to create a logistic regression discriminant function to compute the probability of an infection. A study involving 16 patients and 22 healthy control subjects resulted in a sensitivity of 87.5 % and a specificity of 100 %. The overall accuracy is 94.7 %.

Casalino et al. (2018) [19] introduced a real-time monitoring system for cardiovascular risk detection through the analysis of HR, RR, peripheral oxygen saturation (SpO$_{2}$), and lip color. The measurement hardware consisted of a single standard HD 1080p webcam. Three rPPG signals are computed from the red, green, and blue channels from an ROI on the patient’s forehead and used for determining the HR and RR. SpO$_{2}$ is computed according to [20] via the red and blue rPPG channels. Lip color is clustered into three categories via K-Means clustering [21] and determined via the RGB value of the dominant color. The medical significance of different lip colors with respect to cardiovascular risk is provided by a medical professional. After determining the four vital parameters, the rules for the fuzzy logic [22] are set with the help of a medical professional. The fuzzy rules classify cardiovascular risk into four categories, namely: low, medium, high, and very high. For the evaluation of the risk assessment, a data set consisting of measurements of 116 patients is generated. The risk assessment evaluation resulted in an overall accuracy of 69 %.

Jiang et al. (2020) [23] reported an early respiratory infection screening method for persons wearing a protective face mask by the use of IRT. The measurement hardware consists of an Android smartphone and a FLIR ONE Pro smartphone plug-in, a device that contains a thermal and an RGB camera with a resolution of 1.5 MP. Breathing patterns are computed by analyzing facial temperature variations obtained via the IRT camera. The collected raw breathing signal is fed into a bidirectional Gate Recurrent Unit (BiGRU) [24] neural network with an additional attention layer [25]. The GRU neural network is a simplified version of Long–Short-Term Memory (LSTM), which is found to often outperform LSTM networks with few input data [24]. Bidirectional networks have the structural advantage with which they can further strengthen the correlation between the context of the sequence [26]. Lastly, the attention layer does estimations of all outputs to find the most important ones. The BiGRU-AT neural network classifier is used to classify breathing patterns into normal and abnormal. The model is trained and evaluated with data from 73 patients and a total of 4217 measurements. The total accuracy of the model is 83.7 %.

Negishi et al. (2020) [27] presented succeeding research to the previously introduced work from Sun et al. (2017) [17]. While the hardware remains unchanged, the algorithm is adapted, a new patient study is conducted, and new results are presented. The measured vital signs remain HR, RR, and, BT. For the measurement of HR, not only the green channel but also the red and the blue channels are used. For the measurement of RR, besides the nasal, the oral area is included as well. The estimation of BT remains unchanged. The classification model is adapted as well. A support vector machine (SVM) model is introduced with the three vital signs as input values. The evaluation included 28 patients and 22 healthy control subjects, resulting in a sensitivity of 85.7 %, a specificity of 90.1 %, and an overall accuracy of 88.0 %.

Gastel et al. (2021) [18] introduced a multi-vital sign monitoring system for supporting the diagnosis of Obstructive sleep apnea (OSA) [28] in sleep clinics. The measurement hardware consisted of three identical monochromatic cameras with different optical bandpass filters in the near-infrared (NIR) spectrum, allowing spectral selectivity in the NIR spectrum. In addition, a wide-band NIR light source with a visible light-blocking filter is used. The multi-parametric sleep analysis, polysomnography, is used as a reference system and the measurements are evaluated by experienced sleep clinicians according to the standard provided by the American Academy of Sleep Medicine (AASM) [29]. The work introduces the measurement of HR, RR, and SpO$_{2}$. The approach for determining the three vital signs is presented in a previous publication of the same research team in [30]. The study includes eight patients and 46.5 h of video recordings. The measurement of HR and RR have a deviation of under two beats per minute (BPM) and under two respiration per minute (RPM) for 91 % of the measurement duration. The SpO$_{2}$ measurement shows a deviation of up to 4 % within 89 % of the measurement duration. The authors state that the findings may be used as a surrogate measurement system during a PSG to decrease the number of contact-based sensors in the procedure.

### Study comparison

The summary of all five included studies is given together with references to previous works or cited works from other authors. The summary shall give the reader a short and clear insight into each included work itself along with specific details which are relevant to this review.

**Fig. 2:**
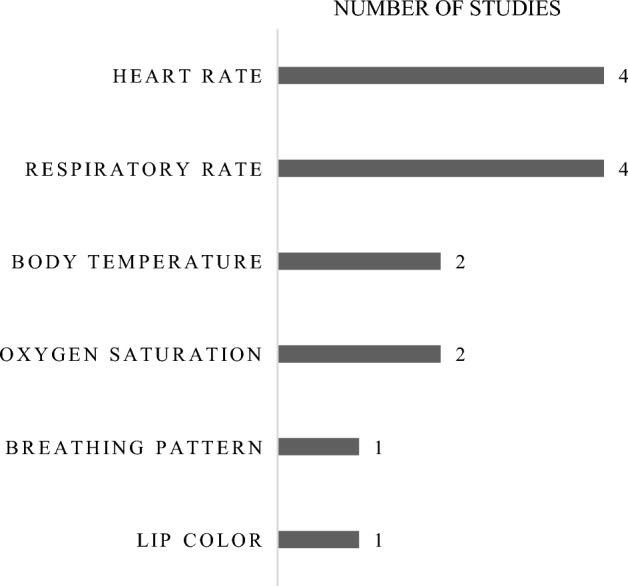
Count of occurrences of different vital sign measurements in the selected studies

A summary of relevant characteristics of all included studies is provided in Tables 1, 2 and 3. In Table 1, a comparison is given regarding the origins of the work, the diagnosis goals, and the specific vital signs which are measured. In Table 2, a comparison regarding the size of the study sample, the way the reference measurements are conducted, the classification model for the diagnosis, and the accuracy of the classification model is given. In Table 3, a comparison regarding the measurement hardware is given, providing details on the optical sensors, the number of analyzed optical channels, and the source of lighting. A summary of the occurrence of different vital signs involved in the diagnosis model is given in Fig. 2. Further discussions on the results of the comparison are provided in section .

**Table 1:** Comparison of the diagnosis goals and measured vital signs within included publications

Article	Publication year	Country	Diagnosis goal	Vital signs
Sun et al. [17]	2017	Japan	Seasonal influenza	HR, RR, BT
Casalino et al. [19]	2018	Italy	Risk of cardiovascular disease	HR, RR, SpO$_{2}$,Lip color
Jiang et al. [23]	2020	China	Early respiratory infection (on the example of COVID-19)	Breathing pattern
Negishi et al. [27]	2020	Japan	Seasonal influenza	HR, RR, BT
Gastel et al. [18]	2021	Netherlands	Support for obstructive sleep apnea diagnosis	HR, RR, SpO$_{2}$

**Table 2:** Comparison of the patient studies and diagnosis models within included publications

Article	Study size	Reference measurement	Diagnosis model	Model accuracy (%)
Sun et al. [17]	38 patients by doctor	Physical examination regression	Logistic	94.7
Casalino et al. [19]	116 patients by doctor	Physical examination	Fuzzy logic	69.0
Jiang et al. [23]	50 patients	Physical examination by doctor	BiGRU-AT neural network	83.7
Negishi et al.[27]	50 patients	Physical examination by doctor	Support vector machine	88.0
Gastel et al. [18]	8 patients	Polysomnography	n/a	n/a

**Table 3:** Comparison of the measurement hardware within included publications

Article	Sensors & resolution	No. of channels	Source of lighting
Sun et al.[17]	Nippon Avionics TVS-500EXLVIRT IRT and RGB 0.3 MP	2 (G & IRT)	Ambient light
Casalino et al.[19]	Microsoft LifeCam HD 1080p RGB Webcam 2.1 MP	3 (R, G & B)	2 x 18 LED strips 12 V, 6 W, 0.5 A,120$^{\circ }$ beam angle
Jiang et al. [23]	FLIR One Pro 1.5 MP IRT & 1.5 MP RGB	4 (R, G, B & IRT)	Ambient light
Negishi et al. [27]	Nippon Avionics TVS-500EXLVIRT IRT and RGB 0.3 MP	4 (R, G, B & IRT)	Ambient light
Gastel et al. [18]	3 x Allied Vision Manta G283B NIR Mono 2.8 MP	3 (3 x NIR Mono)	Exo Terra Night Heat Lamps

### Risk of bias in studies

There is no evident risk of bias in the selection process of studies for this systematic review since none of the authors are or were affiliated with any of the authors or institutions listed in the selected studies. To reduce the risk of bias in the study selection process, two reviewers independently screened the results of the database searches, as described in the subsection on the selection process. Furthermore, the management of the systematic review process is done with the automated online tool Rayyan [31] for keeping a study selection protocol and documenting the decisions of both reviews concerning the eligibility of screened studies. Disagreements in the decisions regarding the study inclusion process between the authors were resolved by discussion until a consensus was reached.

## Discussion

The focus of this review are research studies that propose fully automatic patient diagnosis models based on contactless optical vital parameter measurements. Considering that only five studies met the proposed eligibility criteria, it is evident that this field is highly under-researched. Furthermore, by observing the publication years, it can be seen that the oldest article is published only five years ago, while the youngest one is published just one year ago. This shows that this field is potentially evolving and that the trend is likely to go upward in the years to come. This forecast is supported by the fact that the number of research studies in the field of contactless optical vital sign measurement and monitoring is a highly investigated topic at the moment [14, 15]. Furthermore, multiple reports [32, 33] show that the COVID-19 pandemic [34] increased the global interest in contactless monitoring research. Supporting this report is the fact that three out of the five included studies are designed to detect persons at risk of an infectious disease [17, 23, 27]. Two of the three are specifically designed to detect people with the risk of a COVID-19 infection [23, 27]. One study [23] considers even the infection risk assessment of persons wearing a face mask, a very common means of decreasing the risk of viral transmission.

The remaining two studies do not share the same diagnosis goal. One of the studies [19] deals with the risk prediction of cardiovascular diseases, while the second study [18] deals with supporting the diagnosis of the sleep disorder OSA. The variety of investigated use cases emphasizes the versatility of contactless optical monitoring and its usability in a wide range of diagnosis scenarios.

A high dissimilarity is present in the selection of the optical measurement equipment. The range begins with consumer-level webcams in [19] and reaches high-quality industrial cameras in [18]. Furthermore, the number and spectra of optical channels are likewise diverse (see Table 3). A possible trend is found in the latest three studies [18, 23, 27], in which the infrared (both NIR and FIR) spectrum is given a higher significance. This trend can be explained by considering the following points: (1) the measurement of relative temperature variations in the nasal and oral regions via infrared thermography has shown good results in terms of RR and breathing pattern analysis [27, 35, 36]; and (2) since it is not practicable to use visible light sensors for the measurement of vital signs during sleep, the use of NIR cameras has proven to be a good alternative [18, 37–39].

A further dissimilarity in the selected studies are the selected classification algorithms. Out of the four studies [17, 19, 23, 27] which present a final classification algorithm, every study presents a different model. It is noted again that one included study [18] does not include a final classification and is hence not regarded at this point. The classification algorithms range from well-established methods, such as logistic regression in [17], fuzzy logic in [19] and support vector machine in [27], up to modern ML classifiers such as BiGRU-AT neural networks in [23].

It is further observed that one study [23] has a clear dissimilarity to the other four. While the other four studies follow the following steps: (1) determine values of vital signs individually; and (2) feed the values of the vital signs into the classifier, the study in [23] introduced a different approach. This study is the only one not to determine the values of vital signs (HR, RR, or BT), but rather analyze the breathing patterns to win additional information in the classification process. This is the reason for selecting the BiGRU-AT neural network classifier since this architecture is designed for dealing with patterns and time-series data [24]. The fact that the raw time-series are fed into the classifier directly leads to a high number of input values. This causes high computation time and memory requirements. Furthermore, various publications have demonstrated that classification accuracy can be improved by introducing a feature extraction level before the classifier. Introducing this additional stage can not only result in higher classification accuracy but also in lower computation time and memory requirements due to the lower number of inputs to the classifier. This improvement was demonstrated in several biomedical engineering topics, such as in [40] for seizure detection via EEG analysis, and in [41] for multiple sclerosis classification via gait analysis, but also in other fields, such as in [42] for fault diagnosis in gearboxes. Therefore, the introduction of a feature extraction level in the scope of contactless patient diagnostics has great potential to enhance classification accuracy and decrease computation time and needs to be investigated in the future.

The included studies are highly diversified among all observed parameters. Therefore, the authors do not see a statistical evaluation and scoring among the studies as reasonable. Hence, the statistical comparison between the studies remains on the comparisons in Tables 1, 2 and 3 and in Fig. 2.

Taking into account the reports which show growing global interest in contactless patient monitoring [32, 33], we see high potential for this topic and believe that the topic of contactless optical diagnostics will be stronger investigated in the future. From our point of view, there are several points which remain insufficiently researched and which should be considered in future research, including: (1) a higher focus on systematic clinical evaluation, because a higher number of clinical trials and patient studies will generate more data, which will have the potential to improve the diagnosis models; (2) introduction of new and further studies in the already introduced use cases, since we observed only three separate use cases in the included publications, whereas contactless monitoring methods may be applied to a vast amount of use cases in patient monitoring; and (3) the extraction and analysis of features in biosignals prior to the classification and higher integration of expert medical knowledge into the algorithm design, since classification models of a smaller, however more purposeful amount of input data, will decrease the computation complexity and can lead to higher model accuracy [40–43].

## Conclusion

The low yield of only five studies in the study selection process shows a large research gap in the field of fully automated contactless optical patient diagnostics. A high heterogeneity is observed among the included studies, ranging from the selection of measurement hardware to the implementation of classification algorithms. Nevertheless, the promising results presented in the included studies along with the reports [32, 33] showing a growing interest in contactless patient monitoring prove the potential of this area and we expect that this area will gain more attention in the future.

## Methods

### Protocol

This systematic review is performed in accordance with the guidelines of the Preferred Reporting Items for Systematic Reviews and Meta-Analyses (PRISMA) [44]. The PRISMA checklist is used to follow and include all relevant and applicable review parameters and for the terminology and definition of review items and terms.

### Eligibility criteria

In order for a study to be eligible for this review, the following criteria must be fulfilled: (1) use of solely contactless measurement technologies for the estimation of vital signs and/or other relevant health parameters; (2) exclusive use of optical sensors, ranging from commercially available webcams to industrial level cameras and from visible light sensors to infrared thermography (IRT); (3) a diagnosis of the patient condition is obtained; (4) measurement is compared with a reference medical device or with the diagnosis of a medical professional, and (5) publication is written in English.

### Information sources and search strategy

Research articles published in five digital databases from inception until July 2022 are included in this systematic review. The screened databases include Google Scholar, IEEE Xplore, PubMed, MDPI, and arXiv. The structure of the keyword search is as follows [acute diagnosis OR real-time diagnosis OR rapid diagnosis OR diagnosis] AND [contactless OR optical] AND [vital signs OR heart rate OR respiratory rate OR body temperature OR oxygen saturation]. Bibliographies of eligible articles are screened for missed publications.

### Selection process

Two reviewers generated a list of keywords and a list of databases to include in the selection process. These two lists are presented in the previous subsection. Both reviewers individually conducted the database searches, screened titles and abstracts, and selected articles for further eligibility assessment. The selected articles are added to and further managed by both reviewers through Rayyan [31], an online automatic tool for collaboration on systematic reviews. All of the selected articles for eligibility assessment are independently reviewed by both reviewers and recommended for inclusion, potential inclusion, or exclusion from the study. The final selection of eligible articles for the study is done by the mutual consent of the two reviewers.

### Data collection process

The data sought in the articles includes: (1) information about the hardware setup, including the sensors, sources of lighting, number of optical channels, as well as other setup components; (2) information on the data collection, including the data gathering from the optical measurement system, as well as the data gathering from the reference measurement system; (3) information about the diagnosis goal; (4) information about the algorithm and classification, including the vital signs and other patient parameters that are measured, the algorithms behind the measurements, the classification method and the structure of the classification model; and (5) size of the study and study results.

## Supplementary Information

**Additional file 1.** Protocol of records identification, screening and decisions.

## Data Availability

The protocol and results of the database identification, screening, and decisions are included in the supplementary material. Further inquiries can be directed to the corresponding author.
