# Supplementary material for: Current methods for contactless optical patient diagnosis: a systematic review
Source: Biomed Eng Online. 2023 Jun 17;22:61. doi: 10.1186/s12938-023-01125-8 (PMC10276455; doi:10.1186/s12938-023-01125-8)
Supplement: Supplementary file 1 — Additional file 1. Protocol of records identification, screening and decisions. [file 12938_2023_1125_MOESM1_ESM.pdf]

Protocol of records identification, screening and decisions

Database search

Database	No. of hits
Google Scholar	9837
PubMed	329
MDPI	95
IEEE Xplore	54
arXiv	11

Screening

Step in screening process	No. of records
Total	10326
Duplicates	3089
Removed for other reasons	6819
Records to screen	418
Records excluded after initial screening	381
Full text sought for retrieval	37
Retrieved full text	35
Not retrieved full text	2
Incorrect study design	21
Contact-based method	5
Non-optical method	4
Included in the review	5
